# Supplementary material for: Genetic associations with human longevity are enriched for oncogenic genes
Source: medRxiv. 2024 Aug 1:2024.07.30.24311226. Preprint. [Version 1] doi: 10.1101/2024.07.30.24311226 (PMC11312667; doi:10.1101/2024.07.30.24311226)
Supplement: Supplement 1 [file NIHPP2024.07.30.24311226v1-supplement-1.pdf]

medRxiv preprint doi: https://doi.org/10.1101/2024.07.30.24311226; this version posted August 1, 2024. The copyright holder for this preprint (which was not certified by peer review) is the author/funder, who has granted medRxiv a license to display the preprint in perpetuity.
It is made available under a CC-BY 4.0 International license .

Supplementary Figure 1. Phenome-wide association of rs13190937 on *ZSCAN23*. This analysis is based on PheWeb (https://pheweb.org/UKB-Neale/).

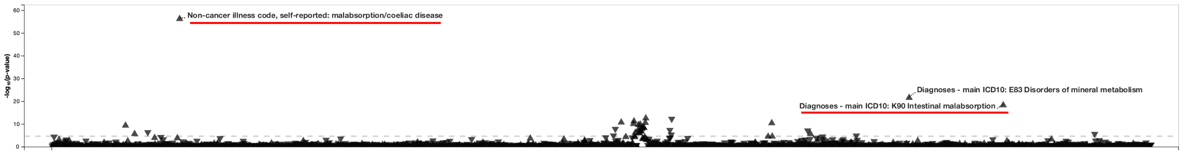

medRxiv preprint doi: https://doi.org/10.1101/2024.07.30.24311226; this version posted August 1, 2024. The copyright holder for this preprint (which was not certified by peer review) is the author/funder, who has granted medRxiv a license to display the preprint in perpetuity.
It is made available under a CC-BY 4.0 International license .

Supplementary Figure 2. Sex-stratified common variant GWAS of longevity. Manhattan plot in males (A), and females (B). Locuszoom (C) and colocalization (D) plots at the *MUC5B* locus in males, colocalized with *MUC5B* eQTL in lung tissue in GTEx. PP4: posterior probability of colocalization. (E) Phenom-wide association of rs35705950. This analysis is based on PheWeb (https://pheweb.org/UKB-Neale/).

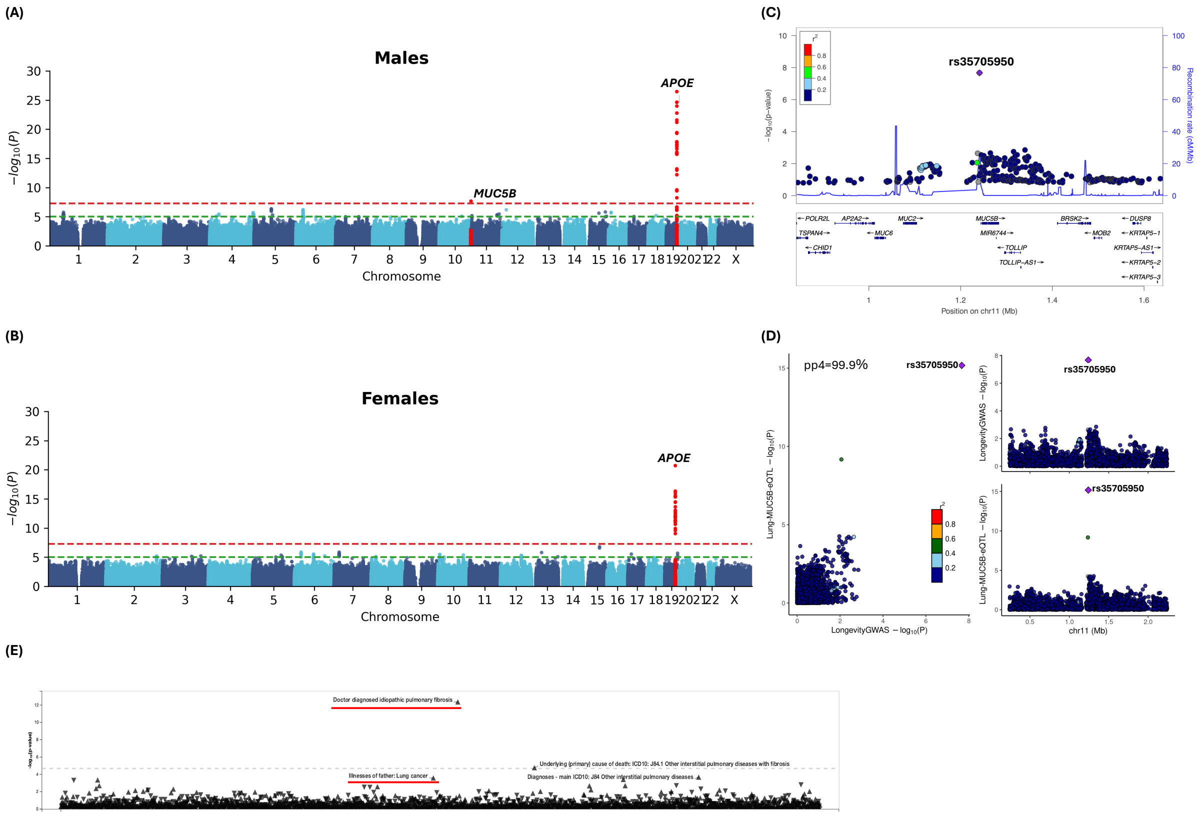

medRxiv preprint doi: https://doi.org/10.1101/2024.07.30.24311226; this version posted August 1, 2024. The copyright holder for this preprint (which was not certified by peer review) is the author/funder, who has granted medRxiv a license to display the preprint in perpetuity.
It is made available under a CC-BY 4.0 International license .

Supplementary Figure 3. Rare variant SKAT-O association with longevity considering 3 categories: Loss-of-function (A), Alpha Missense (B), and REVEL (C). Novel genes are highlighted in red.

(A)

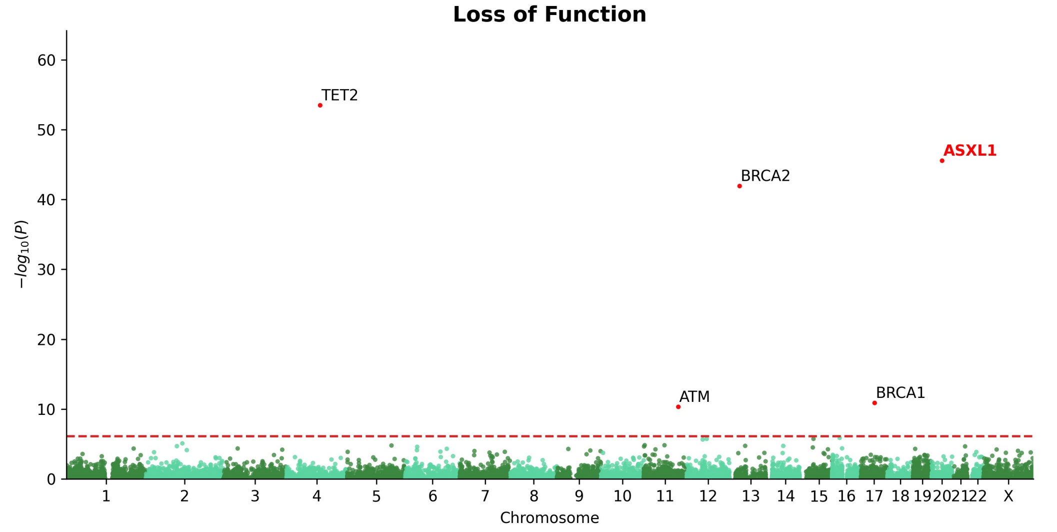

(B)

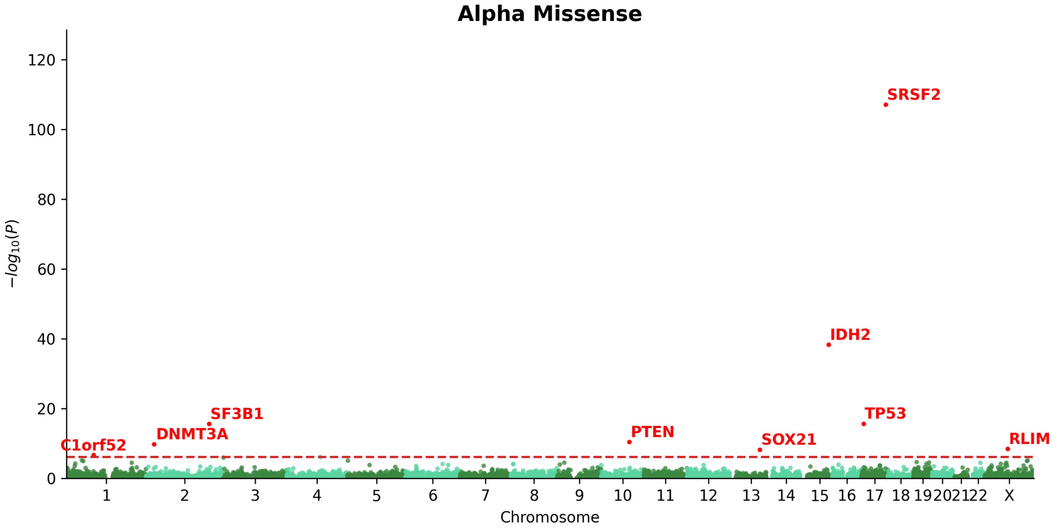

(C)

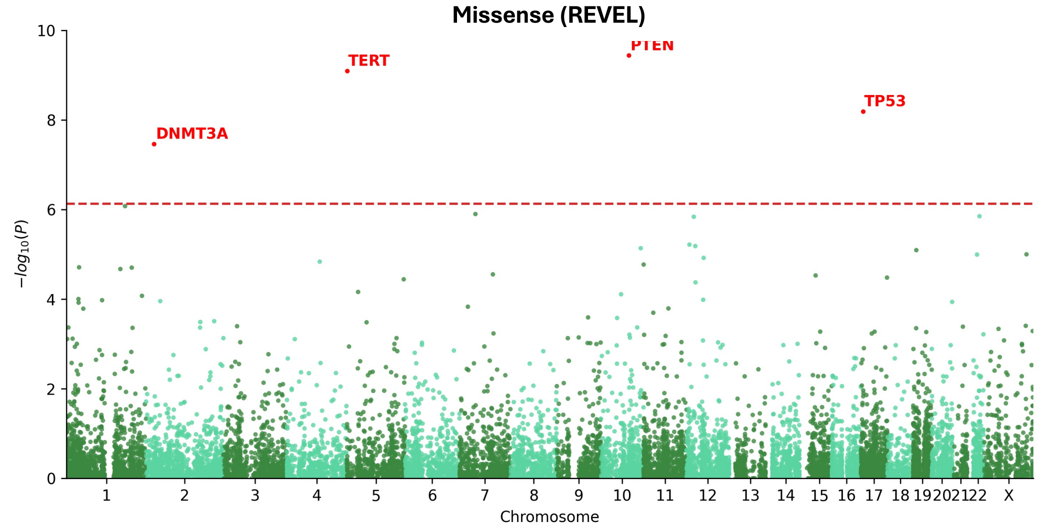

medRxiv preprint doi: https://doi.org/10.1101/2024.07.30.24311226; this version posted August 1, 2024. The copyright holder for this preprint (which was not certified by peer review) is the author/funder, who has granted medRxiv a license to display the preprint in perpetuity.
It is made available under a CC-BY 4.0 International license .

Supplementary Figure 4. Rare variant burden association with longevity considering REVEL pathogenic missense variants. Novel genes are highlighted in red.

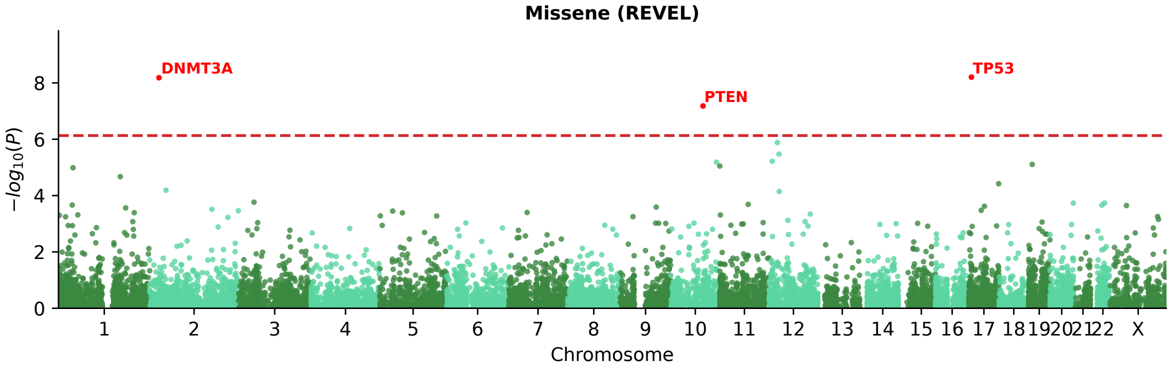

medRxiv preprint doi: https://doi.org/10.1101/2024.07.30.24311226; this version posted August 1, 2024. The copyright holder for this preprint (which was not certified by peer review) is the author/funder, who has granted medRxiv a license to display the preprint in perpetuity.
It is made available under a CC-BY 4.0 International license .

Supplementary Figure 5. Sex-stratified rare variant burden association with longevity considering 3 categories for each sex: Loss-of-function (A), Alpha Missense (B), and REVEL (C). Novel genes are highlighted in red.

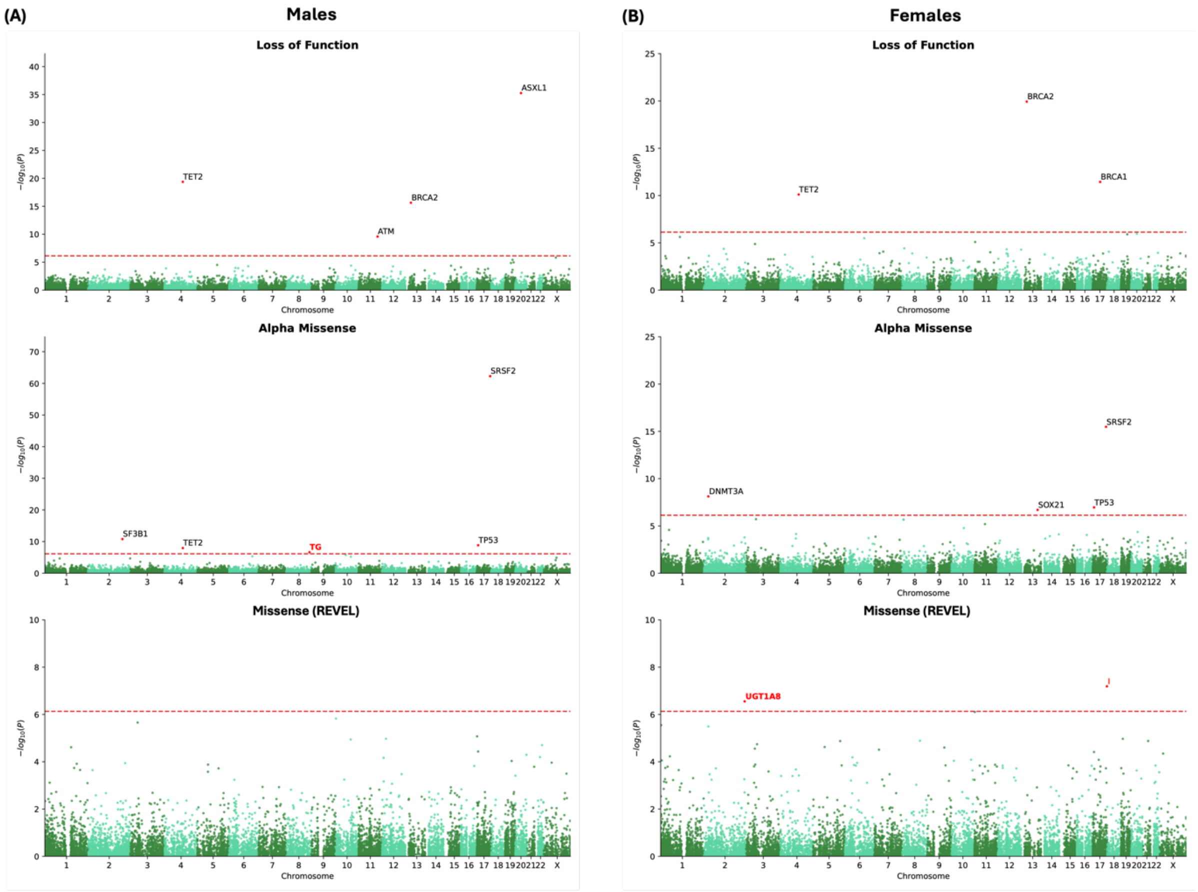

medRxiv preprint doi: https://doi.org/10.1101/2024.07.30.24311226; this version posted August 1, 2024. The copyright holder for this preprint (which was not certified by peer review) is the author/funder, who has granted medRxiv a license to display the preprint in perpetuity.
It is made available under a CC-BY 4.0 International license .

Supplementary Figure 6. Sex-stratified rare variants SKAT-O association with longevity considering 3 categories for each sex: Loss-of-function (A), Alpha Missense (B), and REVEL (C). Novel genes are highlighted in red.

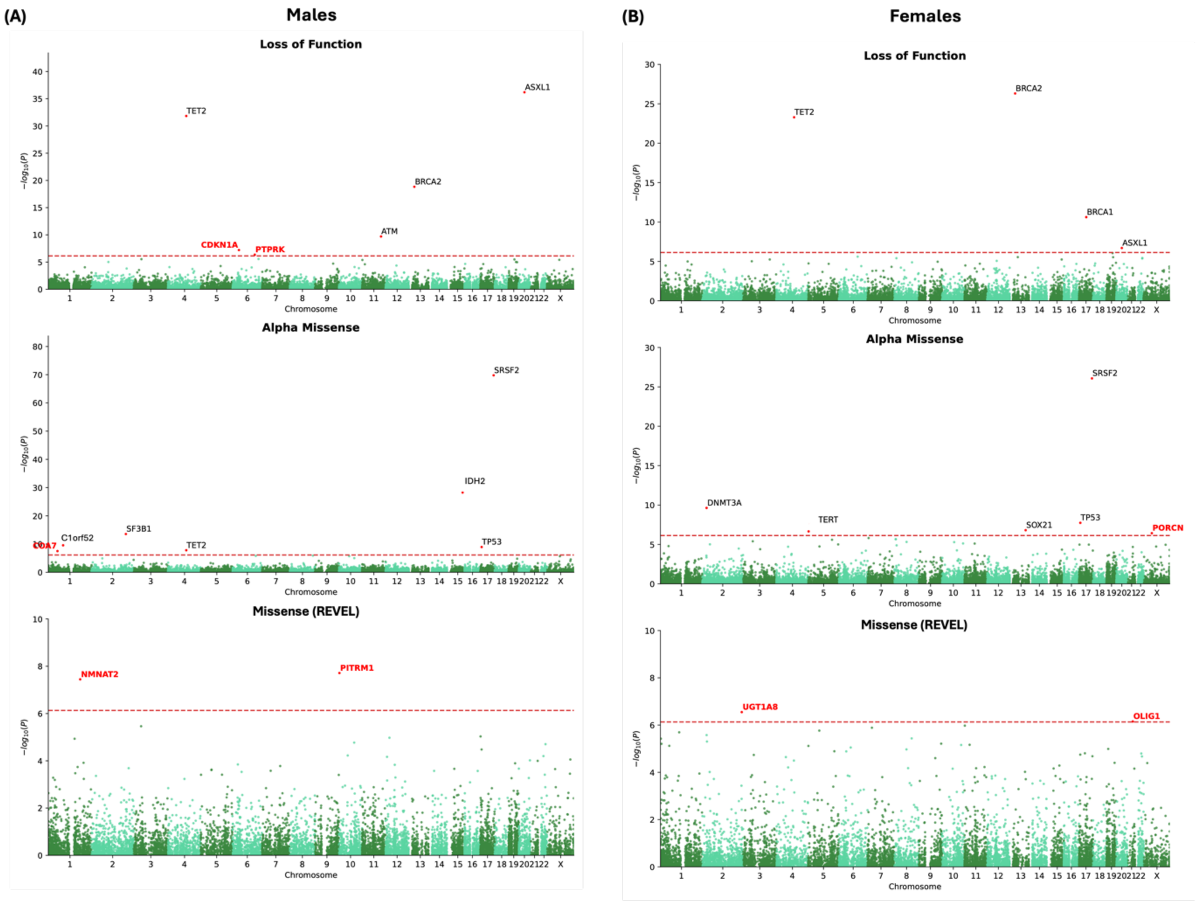

medRxiv preprint doi: https://doi.org/10.1101/2024.07.30.24311226; this version posted August 1, 2024. The copyright holder for this preprint (which was not certified by peer review) is the author/funder, who has granted medRxiv a license to display the preprint in perpetuity.
It is made available under a CC-BY 4.0 International license .

Supplementary Figure 7. Survival curves comparing carriers and non-carriers of variants considered on genes with a significant burden of loss-of-function (*TET2*, *ATM*, *BRCA2* and *BRCA1*) (A), AlphaMissense pathogenic (B) variants (*TET2*), missense variants predicted by REVEL (*DNMT3A*, *PTEN* and *TP53*) (C)

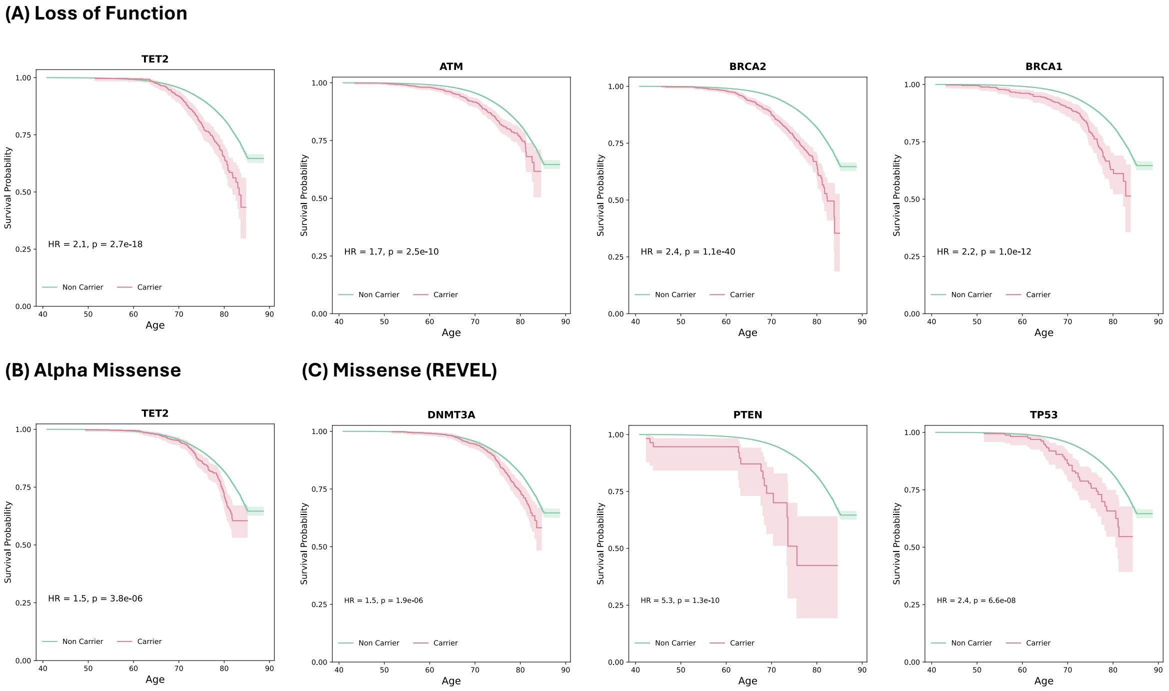

medRxiv preprint doi: https://doi.org/10.1101/2024.07.30.24311226; this version posted August 1, 2024. The copyright holder for this preprint (which was not certified by peer review) is the author/funder, who has granted medRxiv a license to display the preprint in perpetuity.
It is made available under a CC-BY 4.0 International license .

Supplementary Figure 8. Phenome-wide association of the burden of rare variants at the nine novel genes identified in our burden test. Variants considered correspond to loss-of-function and Alpha missense defined variants. P-values less than $1.0\text{x}10^{-50}$ are capped at 50.

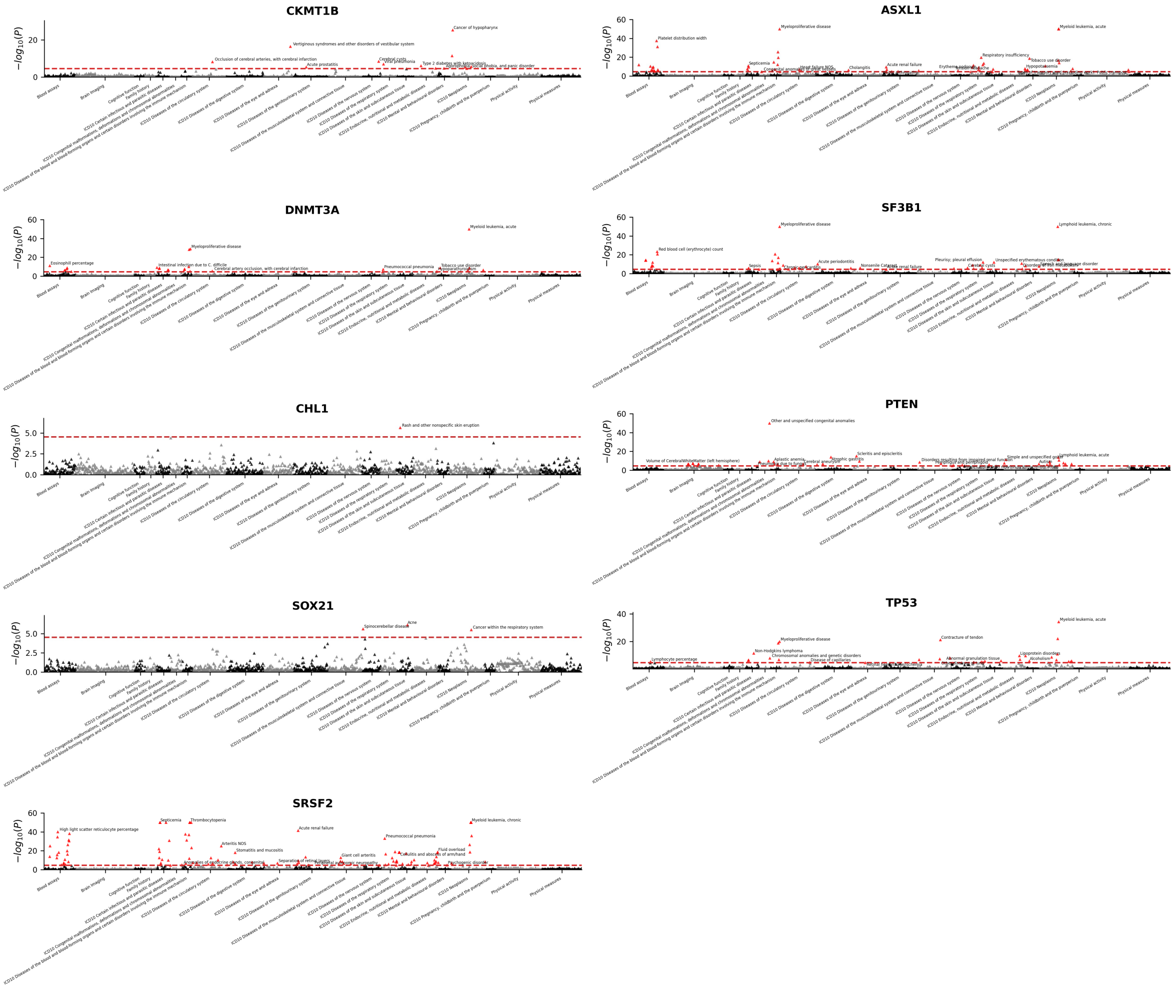

medRxiv preprint doi: https://doi.org/10.1101/2024.07.30.24311226; this version posted August 1, 2024. The copyright holder for this preprint (which was not certified by peer review) is the author/funder, who has granted medRxiv a license to display the preprint in perpetuity.
It is made available under a CC-BY 4.0 International license .

Supplementary Figure 9. Variant allelic fraction distribution per gene for variants considered in each category: Loss-of-function (A) and Alpha Missense (B).

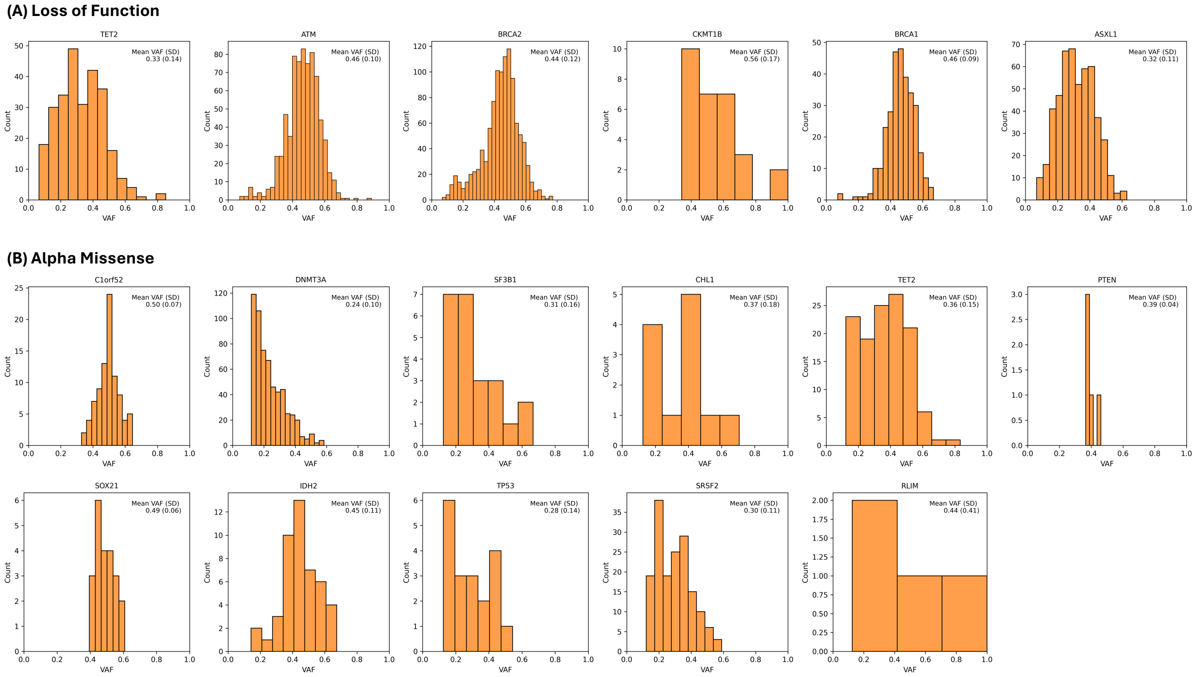

medRxiv preprint doi: https://doi.org/10.1101/2024.07.30.24311226; this version posted August 1, 2024. The copyright holder for this preprint (which was not certified by peer review) is the author/funder, who has granted medRxiv a license to display the preprint in perpetuity.
It is made available under a CC-BY 4.0 International license .

Supplementary Table 1. Demographics of European ancestry in the analyses.

	All			Male			Female		
	**Total**	**Living**	**Deceased**	**Total**	**Living**	**Deceased**	**Total**	**Living**	**Deceased**
N	393,833	358,282	35,551	180,970	159,911	21,059	212,863	198,371	14,492
Last known age	70.8 ± 7.9	70.7 ± 8.0	71.2 ± 7.5	70.9 ± 8.0	70.8 ± 8.1	71.3 ± 7.4	70.7 ± 7.9	70.7 ± 7.9	71.1 ± 7.6
APOE ε4 carrier	113,437 (28.8%)	102,360 (28.6%)	11,077 (31.2%)	52,190 (28.8%)	45,635 (28.5%)	6,555 (31.1%)	61,247 (28.8%)	56,725 (28.6%)	4,522 (31.2%)

medRxiv preprint doi: https://doi.org/10.1101/2024.07.30.24311226; this version posted August 1, 2024. The copyright holder for this preprint (which was not certified by peer review) is the author/funder, who has granted medRxiv a license to display the preprint in perpetuity.
It is made available under a CC-BY 4.0 International license .

Supplementary Table 2. Significant genes for burden and SKAT-O association of rare variants, considering missense variants with REVEL > 75. Gene names in bold font represent novel associations in REVEL.

Variant Class	Chr	Gene	# of variants	# of carriers	Burden *p*-value	SKAT-O *p*-value
REVEL (>75)	2	*DNMT3A*	116	831	6.6×10^{-9}	3.5×10^{-8}
	5	***TERT***	31	60	2.6×10^{-3}	8.1×10^{-10}
	10	*PTEN*	42	45	6.6×10^{-8}	3.6×10^{-10}
	17	*TP53*	47	173	6.2×10^{-9}	6.5×10^{-9}

medRxiv preprint doi: https://doi.org/10.1101/2024.07.30.24311226; this version posted August 1, 2024. The copyright holder for this preprint (which was not certified by peer review) is the author/funder, who has granted medRxiv a license to display the preprint in perpetuity.
It is made available under a CC-BY 4.0 International license .

Supplementary Table 3. Significant genes for burden and SKAT-O association of rare variants in males. Genes in bold font represent novel associations in males.

Variant Class	Chr	Gene	# of variants	# of carriers	Burden *p*-value	SKAT-O *p*-value
LoF	4	*TET2*	162	280	4.1×10^{-20}	1.5×10^{-32}
	6	***CDKN1A***	8	33	1.1×10^{-4}	6.3×10^{-8}
	6	***PTPRK***	26	40	5.9×10^{-3}	4.5×10^{-7}
	11	*ATM*	318	520	2.6×10^{-10}	2.0×10^{-10}
	13	*BRCA2*	172	596	2.3×10^{-16}	1.5×10^{-19}
	20	*ASXL1*	59	347	5.4×10^{-36}	6.4×10^{-37}
Alpha Missense (>70)	1	*C1orf52*	19	76	2.2×10^{-5}	2.7×10^{-10}
	**1**	***COA7***	8	11	1.0×10^{-4}	3.1×10^{-8}
	2	*SF3B1*	43	122	1.6×10^{-11}	2.8×10^{-14}
	4	*TET2*	107	405	1.1×10^{-8}	1.6×10^{-8}
	**8**	***TG***	113	657	2.4×10^{-7}	1.2×10^{-6}
	15	*IDH2*	58	171	2.2×10^{-3}	5.5×10^{-29}
	17	*TP53*	24	48	1.5×10^{-9}	1.1×10^{-9}
	17	*SRSF2*	10	104	5.4×10^{-63}	1.7×10^{-70}
REVEL (>75)	1	***NMNAT2***	21	34	1.2×10^{-4}	1.9×10^{-8}
	10	***PITRM1***	6	10	1.5×10^{-6}	3.6×10^{-8}

medRxiv preprint doi: https://doi.org/10.1101/2024.07.30.24311226; this version posted August 1, 2024. The copyright holder for this preprint (which was not certified by peer review) is the author/funder, who has granted medRxiv a license to display the preprint in perpetuity.
It is made available under a CC-BY 4.0 International license .

Supplementary Table 4. Significant genes for burden and SKAT-O association of rare variants in females. Genes in bold font represent novel associations in females.

Variant Class	Chr	Gene	# of variants	# of carriers	Burden *p*-value	SKAT-O *p*-value
LoF	4	*TET2*	151	283	7.8×10^{-11}	5.1×10^{-24}
	13	*BRCA2*	182	675	1.2×10^{-20}	4.8×10^{-27}
	17	*BRCA1*	80	217	3.6×10^{-12}	2.4×10^{-11}
	20	*ASXL1*	46	186	1.1×10^{-6}	2.0×10^{-7}
Alpha Missense (>70)	2	*DNMT3A*	135	671	7.6×10^{-9}	2.3×10^{-10}
	5	*TERT*	22	27	1.7×10^{-3}	2.2×10^{-7}
	13	*SOX21*	34	251	2.0×10^{-7}	1.6×10^{-7}
	17	*SRSF2*	10	37	3.3×10^{-16}	8.4×10^{-27}
	17	*TP53*	23	52	1.1×10^{-7}	1.8×10^{-8}
	X	***PORCN***	11	32	5.6×10^{-4}	3.7×10^{-7}
REVEL (>75)	2	***UGT1A8***	2	18	2.8×10^{-7}	2.8×10^{-7}
	21	***OLIG1***	7	18	1.3×10^{-5}	7.0×10^{-7}

Supplementary Table 5. Lead variant association per gene among significant genes in the burden and SKAT-O tests. Only significant variant associations with at least 3 minor allele counts per gene are reported in this table.

Variant Class	Chr	Gene	Variant	MA	MAC	AM	HR	*p*-value	Reported
REVEL (>75)	2	*DNMT3A*	rs367909007	G	14	0.983	4.3	1.2×10^{-3}	-
	5	*TERT*	rs1043358053	C	5	0.926	11.9	7.4×10^{-7}	-
	10	*PTEN*	rs587782350	C	3	0.941	20.4	2.6×10^{-3}	-
	17	*TP53*	rs11540652	T	5	0.996	10.0	6.6×10^{-5}	Gastric Cancer [70], Ovarian Cancer [71]

medRxiv preprint doi: https://doi.org/10.1101/2024.07.30.24311226; this version posted August 1, 2024. The copyright holder for this preprint (which was not certified by peer review) is the author/funder, who has granted medRxiv a license to display the preprint in perpetuity.
It is made available under a CC-BY 4.0 International license .

Supplementary Table 6. Mean variant allelic fraction per gene across participants included in the corresponding gene-level Burden/SKAT−O analysis.

Variant Class	Chr	Gene	# of subjects	# of variants	Mean VAF (SD)
LoF	4	*TET2*	266	133	0.33 (0.14)
	11	*ATM*	734	128	0.46 (0.10)
	13	*BRCA2*	1,061	162	0.44 (0.12)
	15	*CKMT1B*	29	8	0.56 (0.17)
	17	*BRCA1*	302	74	0.46 (0.09)
	20	*ASXL1*	502	30	0.32 (0.11)
Alpha Missense	1	*C1orf52*	87	11	0.50 (0.07)
	2	*DNMT3A*	593	33	0.24 (0.10)
	2	*SF3B1*	23	5	0.31 (0.16)
	3	*CHL1*	12	3	0.37 (0.18)
	4	*TET2*	123	41	0.36 (0.15)
	10	*PTEN*	5	3	0.39 (0.04)
	13	*SOX21*	22	6	0.49 (0.06)
	15	*IDH2*	46	14	0.45 (0.11)
	17	*TP53*	19	7	0.28 (0.14)
	17	*SRSF2*	164	6	0.30 (0.11)
	X	*RLIM*	4	2	0.44 (0.41)
